# Simulation of Lower Limb Muscle Activation Using Running Shoes with Different Heel-to-Toe Drops Using Opensim

**DOI:** 10.3390/healthcare11091243

**Published:** 2023-04-26

**Authors:** Wenjing Quan, Linna Gao, Datao Xu, Huiyu Zhou, Tamás Korim, Shirui Shao, Julien S. Baker, Yaodong Gu

**Affiliations:** 1 Faculty of Sports Science, Ningbo University, Ningbo 315211, China; 2 Department of Materials Engineering, Faculty of Engineering, University of Pannonia, H-8201 Veszprem, Hungary; 3 School of Health and Life Sciences, University of the West of Scotland, Glasgow G72 0LH, UK; 4 Centre for Health and Exercise Science Research, Department of Sport, Physical Education and Health, Hong Kong Baptist University, Hong Kong 999077, China

**Keywords:** minimalist running shoes, normal shoes, muscle force, heel-to-toe drop

## Abstract

Background: Although numerous studies have been conducted to investigate the acute effects of shoe drops on running kinematics and kinetic variables, their effects on muscle forces remain unknown. Thus, the primary aim of this study was to compare the muscle force, kinematics, and kinetic variables of habitually rearfoot runners with heel-to-toe drops of negative 8 mm shoes (minimalist shoes) and positive 9 mm shoes (normal shoes) during the running stance phase by using musculoskeletal modeling and simulation techniques. Methods: Experimental data of lower limb kinematics, ground reaction force, and muscle activation from 16 healthy runners with rearfoot strike patterns were collected and analyzed in OpenSim. Using Matlab, the statistical parameter mapping paired t-test was used to compare the joint angle, moment, and muscle force waveform. Results: The results revealed differences in the sagittal ankle and hip angles and sagittal knee moments between the different heel-to-toe drops of running shoes. Specifically, it showed that the negative 8 mm running shoes led to significantly smaller values than the positive 9 mm running shoes in terms of the angle of ankle dorsiflexion, ankle eversion, knee flexion, hip flexion, and hip internal and hip external rotation. The peak ankle dorsiflexion moment, ankle plantarflexion moment, ankle eversion moment, knee flexion moment, knee abduction moment, and knee internal rotation also decreased obviously with the minimalist running shoes, while the lateral gastrocnemius, Achilleas tendon, and extensor hallucis longus muscles were obviously greater in the minimalist shoes compared to normal shoes. The vastus medialis, vastus lateralis and extensor digitorum longus muscles force were smaller in the minimalist shoes. Conclusions: Runners may shift to a midfoot strike pattern when wearing negative running shoes. High muscle forces in the gastrocnemius lateral, Achilleas tendon, and flexor hallucis longus muscles may also indicate an increased risk of Achilleas tendonitis and ankle flexor injuries.

## 1. Introduction

Running has become one of the most popular forms of exercise globally [1,2]. Running is associated with preventing obesity and cardiovascular disease [1,3]. However, running provides us with the highest incidence of injuries. The incidence of running injuries has been reported to have increased from 19.4 to 79.3% [4]. Several studies have found that running injuries are mainly lower extremity injuries for professional and amateur runners, especially the knee joint and anterior knee (such as patellofemoral joint pain) [5,6]. Other common injuries include tibial and fibular periostitis, Achilles tendonitis, plantar fasciitis, and iliotibial band syndrome [4,7]. Numerous factors might influence running injuries, including the strike pattern, footwear, and high ground reaction forces during running [8,9,10]. The relationship between biomechanical factors and the risk of running injuries has led to several methods to reduce running injury rate, such as modifying running strike patterns and limiting the running distance [11].

According to foot strike pattern, there are three main types of running patterns: rearfoot strike (RFS), midfoot strike (MFS), and forefoot strike (FFS) [12]. Research relating to running strike patterns found that 75% of runners were habitually rearfoot-strikers [13]. Strike pattern technologies were also associated with running injuries. In RFS running, there may be increases in the loading rate of the impact and knee power. However, the forefoot strike pattern would increase the ankle power and Achilles tendon force [14]. In addition, a good pair of running shoes is essential and desirable for runners. In the past 30 years, running shoes with cushioned and comfortable features have reduced running injuries [15]. Moreover, a previous study has reported that changing the running strike pattern and controlling the running distance might induce running overuse injuries [11]. Thus, many shoe manufacturers have also begun to focus on barefoot running, in which Vibram Five Fingers, Nike Free, and minimalist shod were produced using the forefoot strike pattern [16,17]. Minimalist shoes have features such as low drop, ultra-light, high bend, low cushioning, and so on [18]. Furthermore, a previous study has shown that participants running with minimalist shod may decrease the knee extension moment and patellofemoral joint contact force, enhancing the foot muscle force [19]. A meta-analysis comparing the running economy of running in barefoot, minimalist, and standard running shoes found that running in barefoot [17] minimalist shoes require less oxygen utilization [20]. Nevertheless, much research in recent years has reported that minimalist running shoes may increase the loading rate and ankle joint load [21,22].

The heel-to-toe drop is defined as the difference between the height of the heel and the forefoot of a shoe. The heel-to-toe drop is an essential factor related to the risk of running injuries. It has been reported that lower heel-to-toe drop shoes of 0 mm and 6 mm instead of 10 mm were less likely to injure occasional runners than regular runners [23]. Therefore, the heel-toe drop is a vital parameter for runners to prevent running injuries. Increased shoe heel-to-toe drop might result in a rearfoot strike running gait [24,25]. Horvais et al. compared the effects of heel height and heel-to-toe drop difference on the foot-strike pattern and running kinematics, in which the foot-strike pattern was associated with the lower heel-toe drop. The results demonstrated a positive correlation between the drop of shoes and running contact time during the running stance phase. With the lower shoe drop, the foot angle at contact and contact time were decreased [24]. Chambon et al. reported that running with lower heel-toe drop shoes may influence the foot strike pattern. For example, running in shoes with 0 mm might lead the rearfoot strike into a midfoot strike pattern [26]. Furthermore, the negative heel-to-toe drop running shoes will change the strike pattern into a midfoot strike pattern during the running stance [27].

Electromyography is an essential parameter for characterizing muscle activity during running. Lower limb muscles may provide proper joint alignment, stability, stiffness and propulsion to propel the body forward while running. Yong et al. concluded that RMS (root mean square) activity in the tibialis anterior in FFS runners was considerably reduced compared to RFS runners during the final swing phase. In FFS runners, on the other hand, the medial and lateral gastrocnemius demonstrated much larger RMS (root mean square) activity during the terminal swing phase [28]. Fernandes Ervilha et al. showed that the iEMG (EMG intensity) of the TA (tibialis anterior) was increased when running in shoes utilizing rearfoot strike patterns than in shoes of forefoot strike pattern and barefoot running. Moreover, the iEMG (EMG intensity) of SO (soleus) and GM (gastrocnemius medialis) were significantly smaller when running in the shoes using rearfoot strike patterns [29]. The previous study found that plantar flexor muscles were stimulated 11% earlier and for 10% longer in FFS runners than in RFS runners [30]. Previous research has also found modest differences in muscle activation while running barefoot versus shod. In barefoot runners, the medialis gastrocnemius, lateralis gastrocnemius, and soleus muscle activity were significantly increased [31]. Numerous studies focus on muscle activities; nevertheless, there is no study on muscle force evaluation for minimalist shoes and traditional shoes.

There have also been no previous studies investigating how heel-to-toe drop affects lower extremity muscle force. Thus, the main aim of this study was to use musculoskeletal modeling and simulation techniques to compare the muscle force, kinematics, and kinetic variables of habitually rearfoot runners while wearing the heel-to-toe drop of negative 8 mm shoes (minimalist shoes) or the heel-to-toe drop of positive 9 mm shoes (normal shoes) during the running stance phase. This study aimed to focus on the immediate effect of kinematic and kinetic variables during the running stance with different heel-to-toe drop shoe conditions. It was hypothesized that the plantar flexors (gastrocnemius medialis /lateralis, and soleus) and Achilles tendon force might increase when running in shoes with a heel-to-toe drop of negative 8 mm (minimalist shoes). It was also hypothesized that the ankle, knee kinematics, and kinetic variables might change when running in shoes with a heel-to-toe drop of negative 8 mm (minimalist shoes) compared to running in shoes with a heel-to-toe drop of positive 9 mm (normal shoes).

## 2. Materials and Methods

### 2.1. Participants

Before the test, the sample size was estimated using G*Power (Version 3.1.9.7). Taking into account the effect size of 0.4, the power value of 0.8, and the alpha level of 0.05 [27], a priori power analysis has shown that a sample size of 13 was enough to adequately power this study [27]. We therefore recruited 16 healthy recreational male runners (age: 26 ± 2.0, weight: 73.5 ± 4.6, height: 175.8 ± 0.5 cm) to participate in this study. The following conditions had to be met for runners to qualify as recreational: running using a rearfoot striking pattern and running 2 to 5 km a week [32]. All subjects having the target foot length of US size 9 (±0.5) and self-reported as right leg dominant were included. None of the participants had any lower limb injuries in the past six months. All the participants had no prior experience with minimalist running shoes and the negative value of running shoes. To avoid subjects changing their strike pattern during the running test, all the participants were not informed of the test’s purpose before the study and were only informed about the experimental methodology. This study was approved by Ningbo University Health Research Ethics (protocol code: RAGH 20220116), and prior to the study, all the participants were informed about experimental conditions and provided written consent.

### 2.2. Experimental Shoes Condition

**Figure 1:**
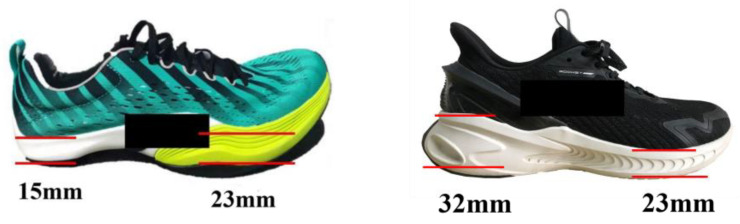
Outlined in this illustration, the left running shoe has the −8 mm HTD (minimalist shoes) and the right running shoe has with 9 mm HTD (normal shoes).

This study used two pairs of running shoes (AT US 9). The difference between the two running shoes was the heel-toe drop (HTD). The heel-to-toe drop in running shoe design is the difference in thickness between the forefoot and heel regions of the sole [33]. Figure 1 showed the test shoes in this study. Heel-to-toe drop differs significantly between regular and negative running shoes, with a −8 mm offset in in negative shoes and 9 mm in normal shoes. It is common for both conventional and negative shoes’ soles to be comprised of EVA foam.

### 2.3. Data Collection

Before the test, all the participants were familiarized with test process. During data collection, running shoe conditions were assigned randomly to the participants. They were asked to make themselves adapt to each running shoes. Then runners were randomized and wore the experience running shoes to run through a 10 m walkway. A device regulating the subjects’ speed was placed on either side of the force platform (smart speed, Fusion Sport Inc. of Burbank, CA, USA). Equipment for assessing speed was located 3.0 m away. Speed was controlled at 3.0 ± 0.5 m/s [32]. Thirty-eight reflective markers (diameter: 14 mm) were attached to the bilaterally lower limbs, torso, and head according to the Opensim Gait 2392 model [34]. The marker coordinates were captured using an eight-camera Vicon motion analysis system (Oxford Metrics Ltd., Oxford, UK) at a frequency of 200 Hz. A force plate (Kistler Type, 9281 B, Kistler Instrument AG, Winterthur, Switzerland) was utilized to collect the ground reaction force (GRF) at 1000 Hz. The markers were placed on: Sternum, R. Acromion, L. Acromion, Toe. Head, R. ASIS, L. ASIS, V. Sacral, Thigh. Upper, R. Thigh. Front, R. Thigh. Rear, R. Knee. Lat, R. Knee. Med, R. Shank. Upper, R. Shank. Front, R. Shank. Rear, R. Ankle. Lat, R. Ankle. Med, R. Heel, R. Midfoot. Sup, R. Midfoot. Lat, R. Toe. Lat, R. Toe. Med, R. Toe. Tip, L. Thigh. Upper, L. Thigh. Front, L. Thigh. Rear, L. Knee. Lat, L. Knee. Med, L. Shank. Upper, L. Shank. Front, L. Shank. Rear, L. Ankle. Lat, L. Ankle. Med, L. Heel, L. Midfoot. Sup, L. Midfoot. Lat, L. Toe. Lat, L. Toe. Med, L. Toe. Tip [35]. All participants completed a static calibration and ran at a pace of 3.0 m/s over a flat runway in a standard sports biomechanics Lab. All subjects were required to run along the walkway with the right foot stepping on the force plate, six successful trials were finally captured. One successful trial was defined as the participant’s right foot running through the whole force plate at the 3.0 ± 0.5 m/s. The surface electromyogram (EMG) wireless 32-channel system (Delsys, Boston, MA, USA) collected participants’ muscle activities during the running phase. Muscle activity included vastus lateralis (VL), vastus medialis (VM), medial gastrocnemius (MG), lateral gastrocnemius (LG), soleus muscle (SL), flexor hallucis longus (FHL), and extensor digitorum longus (EDL) and were collected at a frequency of 1000 Hz [36]. Maximal voluntary contractions (MVC) of the muscles were performed for the normalization of muscle activity (0–100%) following a previously established protocol [37].

### 2.4. Data Processing

Trials were processed using Vicon Nexus 1.8.5 (Vicon, Metrics Ltd., Oxford, UK), identifying anatomical and tracking markers before exporting as C3 D files. For each test, a stance phase is a right foot initial contact on the force plate to the right foot toe-off the force plate [38]. Biomechanical data were processed using Visual 3 D (V6.0, C-Motion, Germantown, MD, USA). A low pass Butterworth filter with cut-off frequencies of 20 Hz (kinetic) and 10 Hz (kinematic) was applied [39]. Then, the joint moment and joint angle parameters were exported. EMG data were band-passed (20–480 Hz), full-wave-rectified, and low-passed at 6 Hz before being amplitude-normalized by the highest signal value across all gait trials [28]. The strike index was calculated as the ratio of the location of the center of pressure at the foot strike to the length of the foot. A strike index of 0–33% indicates a rearfoot striker, a strike index of 34–67% indicates a midfoot striker, and a strike index of 68–100% indicates a forefoot striker [40].

### 2.5. Muscle Force Estimation

**Figure 2:**
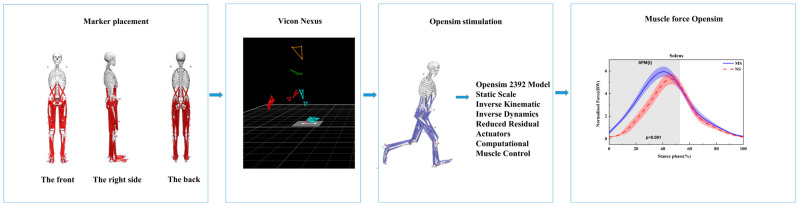
Flowchart of data processing.

The musculoskeletal estimates were completed using the Opensim (vers. 4.3, OpenSim) (Figure 2), which has been used to calculate the muscle force during the running stance [35,36]. The Opensim gait 2392 model was used in our musculoskeletal estimation. The Opensim gait 2392 model including the 10 rigid bodies, 23 degree of freedom, and 92 musculotendon actuators [41]. The entire simulation process in OpenSim consists of model scaling, inverse kinematics (IK), inverse dynamics (ID), the reduced residual calculation (RRA), static optimization, and computational muscle control (CMC) [35]. Firstly, we used the scale tool to fit participant’s anthropometry. Joint angle and joint moment were calculated using the inverse kinematics and inverse dynamics. In order to reduce the residual actuator effort, we used the residual reduction analysis (RRA) to adapt the simulation results. Computational muscle control (CMC) was then used for estimating the muscle force. The Achilles tendon force was finally calculated by adding the medial gastrocnemius, lateral gastrocnemius, and soleus muscle actuator forces [42].

**Figure 3:**
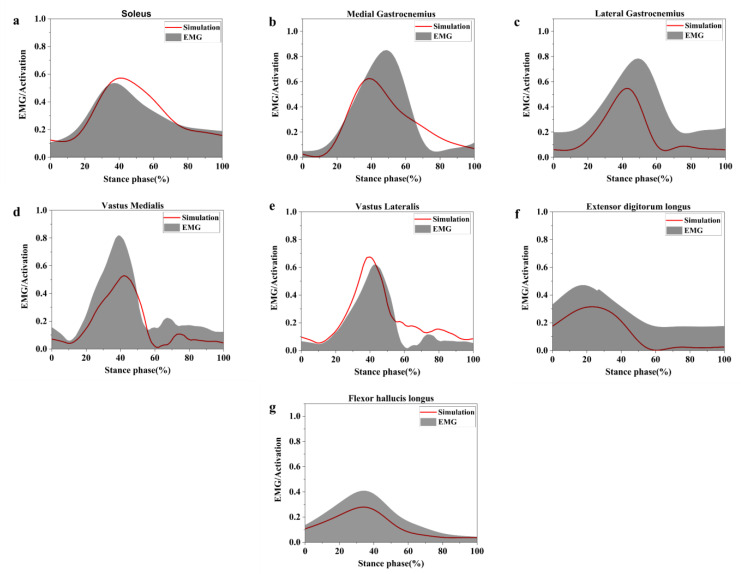
Muscle activation, soleus activation (**a**), medial gastrocnemius activation (**b**), lateral gastrocnemius activation (**c**), Achilles tendon activation (**d**), vastus medialis activation (**e**), vastus lateralis activation (**f**), flexor hallucis longus activation (**g**).

EMG activation variables were compared qualitatively to opensim simulated muscle activations to evaluate opensim model reliability. Figure 3 depicts the comparison results, which indicate that the predicted muscle activation and EMG during the running stance phase were in good agreement.

### 2.6. Statistical Analyses

Statistical analyses were performed in SPSS version 25.0 (SPSS Science, Chicago, IL, USA). Applying the Shapiro–Wilk normality tests to determine the strike index in different running shoe conditions. Paired-sampled *t*-tests were used to compare the strike index. The effect size (Cohen’s d) of the strike index variable was computed in this study. Means and standard deviations of the six valid trials from each subject were calculated for the two different running shoe measurements. The significance level was set at *p* < 0.05. Matlab was used to compare the joint angle, moment, and muscle force waveforms using the open-source, one-dimensional statistical parametric mapping (SPM) program. We created a time series curve with 101 data points using a custom MATLAB script based on data collected during the running phases for statistical parametric mapping (SPM) analysis. The SPM test, which is equivalent to the paired *t*-test, was used [38].

## 3. Results

### 3.1. Joint Kinematics

**Figure 4:**
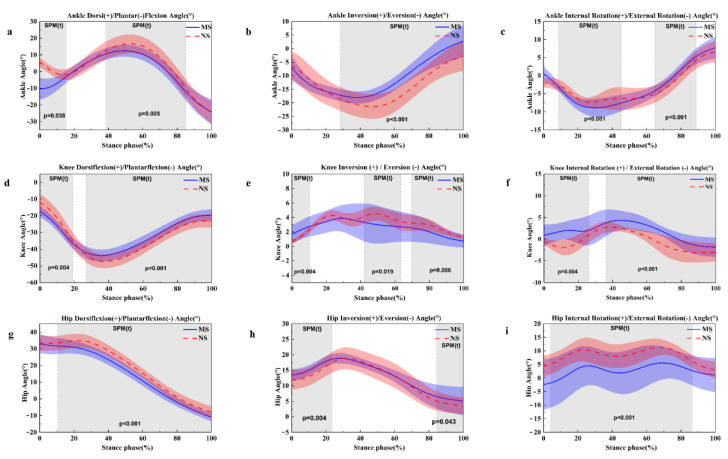
Illustration of the MS and NS lower limb results shows the statistical parametric mapping outputs for the angle of the sagittal ankle (**a**), frontal ankle (**b**), horizontal ankle (**c**), sagittal knee (**d**), frontal knee (**e**), horizontal plane (**f**), and sagittal hip (**g**), frontal hip (**h**) and horizontal hip (**i**) during the running stance phase. MS, minimalist shoes, NS, normal shoes.

Through the strike index calculation, Table 1 presents significant differences between the minimalist shoes and normal shoes. Compared to normal shoes, the foot strike pattern was shifted 21.65% in minimalist shoes. At the ankle joint, dorsiflexion angle increased at 0–15% (*p* = 0.038) and 38–84% (*p* = 0.005) in normal shoes (Figure 4a). The ankle eversion angle was higher during the running stance at 28–100% (*p* < 0.001) with normal shoes (Figure 4b). As for the ankle internal rotation angle, which was decreased at 9–45% (*p* < 0.001) in normal shoes (Figure 4c), at 65–89% (*p* < 0.001), the external rotation angle was significantly increased in normal shoes.

At the knee joint angle, the knee flexion angle decreased at 0–18% (*p* = 0.004) in normal shoes condition (Figure 4d). Compared to minimalist shoes, knee flexion angle was higher during the running stance phase at 27–100% (*p* < 0.001) in normal shoes (Figure 4d). Decreased knee inversion angle was observed at 0–10% (*p* = 0.004) (Figure 4e) in the normal shoes. However, knee inversion angle was increased at 42–63%(*p* = 0.0019) and 69–100% (*p* = 0.006) (Figure 4e) when running in normal shoes condition. However, there was a significantly greater knee internal rotation angle during the running stance at 0–25% (*p* = 0.004) and 36–100% (*p* < 0.001) in normal shoes (Figure 4f).

At the hip joint angle, the dorsiflexion angle increased at 11–100% (*p* < 0.001) in normal shoes (Figure 4g). Compared to minimalist shoes, hip inversion angle at 0–23% (*p* = 0.004) and 84–100% (*p* = 0.043) were significantly smaller in normal shoes (Figure 4h). Increased internal rotation angle was observed during the running stance at 4–88% (*p* < 0.001) in normal shoes (Figure 4i). 

### 3.2. Joint Kinetic

**Figure 5:**
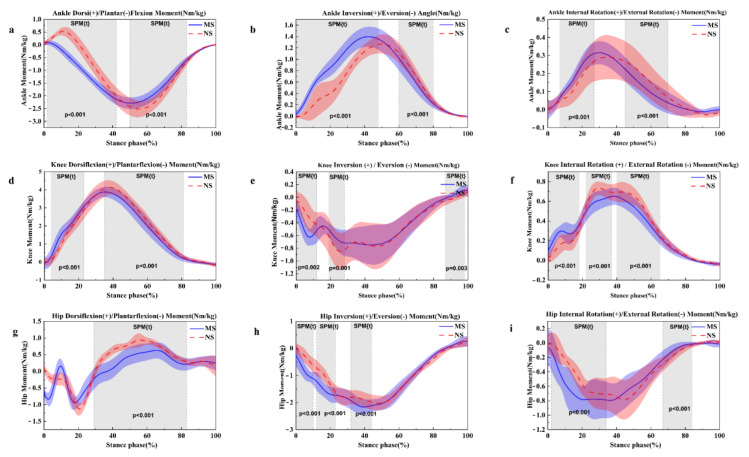
Illustration of the MS and NS lower limb results showing the statistical parametric mapping outputs for the moment of the sagittal ankle (**a**), frontal ankle (**b**), horizontal ankle (**c**), sagittal knee (**d**), frontal knee (**e**), horizontal plane (**f**), sagittal hip (**g**), frontal hip (**h**) and horizontal hip (**i**) during the running stance phase. MS, minimalist shoes, NS, normal shoes.

At the ankle joint moment, dorsiflexion moment and plantarflexion moment were increased at 2–42% (*p* < 0.001) and 50–82% (*p* < 0.001) in normal shoes (Figure 5a). Decreased ankle inversion moment was presented during the running stance at 0–48% (*p* < 0.001) in normal shoes (Figure 5b). However, ankle inversion moment was significantly greater at 60–80% (*p* < 0.001) in normal shoes condition. As for the ankle internal rotation moment, which was decreased at 7–27% (*p* < 0.001) in normal shoes (Figure 5c), at 45–70% (*p* < 0.001), it was significantly greater in normal shoes.

At the knee joint moment, compared to minimalist shoes, the knee flexion moment decreased at 4–23% (*p* < 0.001) (Figure 5d), and the knee flexion moment was significantly increased at 35–81% (*p* < 0.001) in normal shoes. Decreased knee inversion moment was observed at 0–12% (*p* = 0.002) in the normal shoes (Figure 5e). However, compared to minimalist shoes, knee inversion moment was increased at 19–28% (*p* = 0.001) and 87–98% (*p* = 0.003) in normal shoes (Figure 5e). There was a significantly smaller knee internal rotation moment during the running stance at 0–18% (*p* < 0.001) in normal shoes (Figure 5f). The knee internal rotation moment was significantly increased during the running stance at 22–37% (*p* < 0.001) and 40–65% (*p* < 0.001) in normal shoes condition.

At the hip joint moment, dorsiflexion angle was observed to increase the 29–83% (*p* < 0.001) in normal shoes (Figure 5g). There was significant smaller hip eversion angle at 0–10% (*p* < 0.001), 12–23% (*p* < 0.001) and 32–33% (*p* < 0.001) in normal shoes (Figure 5h). Decreased external rotation angle was observed during the running stance at 2–24% (*p* < 0.001) in normal shoes (Figure 5i). Compared to minimalist shoes, hip external rotation angle increased at the running stance at 67–84% (*p* < 0.001) in normal shoes.

### 3.3. Muscle Force

**Figure 6:**
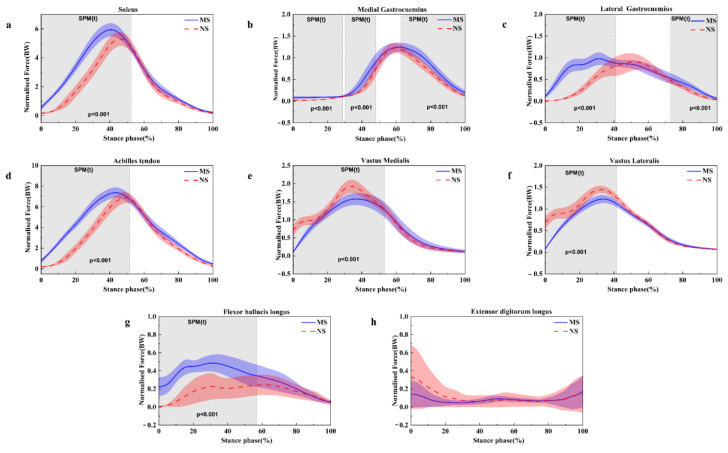
Illustration of the results between the MS and NS lower limb showing the statistical parametric mapping outputs for the soleus force (**a**), medial gastrocnemius force (**b**), lateral gastrocnemius force (**c**), Achilles tendon (**d**), vastus medialis force (**e**), vastus lateralis force (**f**), flexor hallucis longus (**g**) and extensor digitorum longus force (**h**) during the running stance phase. MS, minimalist shoes, NS, normal shoes.

As shown in Figure 6, the characteristic muscle force pattern for running was similar between the normal shoes and minimalist shoes. The largest shift in muscle force across the running stance was observed for the soleus muscle force at 0–48% (*p* < 0.001), medial gastrocnemius force at 0–29% (*p* < 0.001), 30–52% (*p* < 0.001) and 63–100% (*p* < 0.001), which reduced in normal shoes. Simultaneously, lateral gastrocnemius force was also observed to decrease at 0–41% (*p* < 0.001) and 72–100% (*p* < 0.001) during the running stance in normal shoes. There was significantly smaller Achilles tendon force at 0–51% (*p* < 0.001) and flexor hallucis longus force at 0–57% (*p* < 0.001) in normal shoes. However, there was a significantly increased vastus medialis force at 0–47% (*p* < 0.001) and vastus lateral force at 0–42% (*p* < 0.001) in normal shoes.

## 4. Discussion

The main aim of the present study was to compare the joint angle, joint moment, and muscle force in recreational runners while running through the force plate in two different running shoe conditions: a heel-to-toe drop of negative 8 mm shoes (minimalist shoes), and heel-to-toe drop of positive 9 mm shoes (normal shoes) during the running stance phase. We found that when running in minimalist shoes, runners might adjust the running strike pattern from rearfoot strike to forefoot strike. The results of the present study support our hypothesis that plantar flexor and Achilles tendon force might increase when running in minimalist shoes. In addition, the changes in biomechanics parameters were associated with the heel-to-toe drop of footwear.

A previous study showed that a decreased drop in running shoes would alter the running gait into a midfoot strike pattern [26]. Previous research also observed some main differences in kinematics parameters between minimalist and normal running shoes [43]. In general, according to the strike index calculation, all the participants in the present study participants used midfoot strike patterns when running in minimalist shoes during the running stance. Our results were also consistent with the findings of others [27,44]. The lower drops of running shoes increase the ankle’s initial dorsiflexion angle and decrease the contact time during the running stance. Thus, the reduced contact time and initial plantarflexion angle of the joint ankle might cause the strike pattern to change to a midfoot strike pattern. In our study, the heel-to-toe drop of the minimalist shoes was negative 8 mm, which caused the adjustment from the rearfoot strike pattern to the midfoot strike pattern.

Different drops of running shoes were the primary factors influencing the kinematics parameters during the running stance. The minimalist shoes demonstrated less ankle dorsiflexion, ankle eversion, knee flexion, hip flexion, hip internal rotation and hip external rotation angle than the normal shoes. One of the main elements for the kinematic changes of the knee and hip joints is the kinematic changes of the ankle joint during different striking patterns. In the present study, the ankle dorsiflexion angle decreased in minimalist shoes, which also caused the larger plantarflexion angle during the running stance. Due to the lower heel-to-toe drop, the larger ankle plantarflexion angle might decrease the shoe surface angle in the running stance. Compared with runners using the forefoot strike pattern, running using the rearfoot strike pattern condition has a small ankle range motion in the ankle sagittal plane and more significantly greater hip and knee range motion in the sagittal plane [45]. With the decreased heel-to-toe drop, the runners indicated a lower plantar flexion angle and a larger knee range motion to absorb the energy for the toe-off phase [46]. In addition, in FFS running, negative work was greater in the ankle and less in the knee and the forefoot strike pattern decreased during the running stance. In FFS running, the stride length and the center of mass were significantly smaller than the RFS, reducing the work output of the quadriceps [47]. Moreover, the hip flexion and internal rotation angles were more different when running in minimalist shoes and normal shoes. The larger hip flexion and internal rotation angles were observed in the normal shoes during the running stance. A previous study has shown that a greater hip internal rotation angle might increase the iliotibial band friction syndrome [48]. These findings suggested that normal running shoes might increase the running injury rate.

There was a considerable joint moment difference between the minimalist running shoes and the normal running shoes. The ankle joint moment in minimalist shoes was the most noticeable difference in our study. In the minimalist shoes, ankle dorsiflexion, plantar flexion, and eversion moments were dramatically reduced. In the sagittal plane, a significantly larger initial plantarflexion angle was found in the forefoot strike pattern, which might increase the dorsiflexion moment [45]. Similarly, ankle dorsiflexion and rearfoot eversion angles were also observed that can increase in the forefoot strike pattern during the running stance [17,49]. It has been shown that the forefoot strike pattern with shorter stride lengths, larger ankle ROM and knee ROM may increase knee injuries [50]. In addition, the minimalist shoes with the negative heel-to-toe drop need the participant’s triceps to decrease the dorsiflexion angle during the running stance. Then, the Achilles tendon force and ankle plantarflexion moment were all increased [51]. Compared to normal shoes, the knee flexion moment was decreased using the minimalist shoes. Forefoot runners are subjected to less ground reaction and less range motion of lower limb than rearfoot runners; thus, the moment in the sagittal plane of the knee joint is also reduction. Simultaneously, hip abduction and hip external rotation moment was significantly increased using minimalist shoes.

In this study, our findings showed that lateral gastrocnemius force was significantly greater when using minimalist shoes. The lateral and medial gastrocnemius muscle forces have been reported to be greater during barefoot running than in shod running. When running barefoot, the gastrocnemius muscle force would be increased when compared to running shod [52]. Another study also measured the muscle activation and calculated the iEMG of the gastrocnemius and found that the gastrocnemius medialis were significantly smaller when running in the shoes using rearfoot strike patterns [29]. In addition, Divert et al. found values of 24% and 14% greater in the medial and lateral gastrocnemius muscle pre-activation amplitude, respectively, while running barefoot compared to the RFS style shoed condition [53]. In our study, when participants run in minimalist shoes, the negative heel-to-toe drop of running shoes might cause the ankle plantar flexor muscles to enter a pre-activation condition before touching the ground, which increased the lateral gastrocnemius muscle force [30]. As soleus muscle force and Achilles tendon force were increased with the lower heel-to-toe drop of running shoe in the current study. Kulmala et al. reported that data have shown that mid/forefoot runners exhibited larger Achilles tendon force (ATF) and reduced knee loading than rearfoot runners [51]. The results of the current study agreed with previous research. This can be explained due to the negative heel-to-toe drop of running shoes, and participants might use the midfoot strike pattern to contact the ground. The gastrocnemius and soleus muscles would be in pre-activation during the running stance to decrease the impact force, and the soleus force may be greater during the running stance in minimalist shoes. According to a recent study, barefoot and barefoot-inspired running shoes were related to increased Achilles tendon force compared to conventional shoes [54]. As such, the findings suggest that the negative heel-to-toe drop of running shoes may not be adapted for runners who suffer from Achilles tendon injuries.

In addition, the flexor hallucis longus muscle and extensor digitorum longus muscle should be taken into consideration when examining increased ankle plantarflexion angle and moment that normally result from minimalist shoes. The results of our study demonstrated the large flexor hallucis longus muscle force and less extensor digitorum longus muscle force when running in minimalist shoes. During the initial contact ground phase of the forefoot strike pattern, the arch is subjected to a three-point force load, and to better control the deformation of the arch and the elastic potential energy, the muscle group of the foot will have more strength [55,56,57,58]. Therefore, while the increase in flexor hallucis longus muscle was adapted deformation of the arch of the foot and the play of elastic potential energy, minimalist shoes can also enhance the function of the foot.

There are several limitations to our study. First, we only compared the difference of muscle force between minimalist and normal shoes for recreational runners. In the future, muscle simulation should analyze the different running levels of runners, such as experienced runners and novice runners. The small sample size in this study, although powered correctly statistically, may also be a limitation and should be increased in future studies. In addition, the study participants are habitually rearfoot strike runners. Foot strike pattern is an essential factor in influencing running musculoskeletal injuries. Furthermore, the habitual forefoot strike pattern or midfoot strike pattern runners should be considered in future studies. Finally, experimentation investigating female runners would also provide future benefits.

## 5. Conclusions

Shoes with varying heel-to-toe drops might affect biomechanical variables during the running stance. In addition, negative heel-to-toe drop running shoes increase the lateral gastrocnemius, Achilleas tendon, and extensor hallucis longus muscles. This may increase the potential for Achilles tendonitis and ankle flexor injuries. Furthermore, running with low heel-to-toe drop transitions from a rearfoot strike pattern to a midfoot strike pattern. According to the findings of this study, we suggest that athletes without Achilles tendon injuries and strong calf muscles can choose minimalist footwear for running. However, athletes need to pay attention to strengthening exercises to strengthen the foot muscles prior to participation in running activities.

## Figures and Tables

**Table 1:** Strike pattern during the running stance of two running shoes (minimalist vs. normal shoes).

Variables	MS	NS	*p* Value	Cohen’s d
Strike index (%)	47.58 ± 13.39	25.93 ± 12.03	0.001 *	1.70

Note: MS, minimalist shoes; NS, normal shoes; * Significant difference between minimalist shoes and normal shoes (*p* < 0.05).

## Data Availability

The studies involving human participants were reviewed and approved by the Ethics Committee of Ningbo University. The patients/participants provided their written informed consent to participate in this study. The raw data supporting the results of this paper will be made accessible without restriction by the authors.
